# Nerve sheath tumours

**DOI:** 10.1007/s00415-018-9098-y

**Published:** 2018-10-31

**Authors:** James A. Robertson, Emma C. Tallantyre

**Affiliations:** 1 School of Medical Sciences, University of Manchester, Stopford building Oxford Road, Manchester, M13 9PG UK; 2 Department of Neurology, Institute of Psychological Medicine and Clinical Neurosciences, Cardiff University, Cardiff, CF14 4XW UK

## Introduction

Neurofibromas and schwannomas are benign tumours of the peripheral nerve sheath. They are encountered within a wide range of medical specialities and may occur as isolated sporadic lesions or as a key feature of genetic disorders including schwannomatosis, neurofibromatosis type 1 (NF1) and neurofibromatosis type 2 (NF2). Approximately half of cases of NF1 and NF2 are the result of autosomal dominant inheritance of a germline mutation from an affected parent, whilst the remainder are caused by de novo mutations. NF1 is caused by mutations in the *NF1* gene on chromosome 17q11.2, and NF2 is caused by mutation of the *NF2* gene at locus 22q12.2. Despite considerable phenotypic overlap with NF2, schwannomatosis has been shown to a distinct entity with pathological variants in SMARCB1 and LZTR1 genes. NF1 is the most common of the three conditions, with a frequency of 1 in 2500–3000 live births, whereas NF2 and schwannomatosis are considerably less common.

Each of these disorders has a distinct clinical profile. NF1 is predominantly neurocutaneous, characterised by neurofibromas, with concomitant eye and bone manifestations. NF2 is a central disorder, with tumours occurring in cranial and spinal nerves, most commonly bilateral vestibular schwannomas. The characteristic presentation of schwannomatosis is with chronic pain associated with cutaneous and central schwannomas, but without the other features of NF1 or NF2. The nature of these disorders results in overlap in care between neurology, dermatology and genetics and current management is largely symptomatic and surgical, with effective disease modifying treatments remaining elusive.

In this month’s journal club we review two articles relating to these conditions: a study providing insights into the epidemiology of schwannomatosis and NF2, and a phase 1 study examining the use of a selective inhibitor of mitogen-activated protein kinase (MAPK).

## Schwannomatosis: a genetic and epidemiological study

NF2 and schwannomatosis are rare genetic disorders that predispose to the formation of spinal and peripheral nerve schwannomas and have considerable clinical overlap. Although the presence of vestibular schwannomas (VS) in NF2 has previously been considered a distinguishing feature, it is now recognized that LZTR1 schwannomatosis can be associated with VS. The potential for misdiagnosis is clear and population-based epidemiological data scarce, prompting this study.

A national (UK) NF2 and schwannomatosis database was interrogated to identify patients meeting existing diagnostic criteria. All patients underwent DNA sequencing and Multiple Ligation dependent Probe Amplification (MLPA) of the NF2 gene as well as sequencing and MLPA for LZTR1 and SMARCB1 genes (known to be associated with familial schwannomatosis). Tumour samples, where available, were also screened in patients for whom no pathogenic mutation was identified. Epidemiological data were determined both from a highly ascertained regional cohort and within the national population, and clinical phenotypes were compared.

A total of 1192 patients met criteria for NF2 and 399 for schwannomatosis. Within the strict regional boundaries of a highly ascertained nested study there were 95 NF2 and 44 schwannomatosis cases. Regional point prevalence was estimated as 1:50,000 and 1:126,135, respectively. Birth incidence was 1:27,965 for NF2 and 1:68,956 for schwannomatosis. Pathological mutations in LZTR1 were found to account for 16% of the regional cases (and 18.5% of national cases), while mutations in SMARCB1 accounted for 25% of cases regionally (12.5% nationally). A further 4% (3%) of cases had mosaic NF2 and in 50% (67%) no pathogenic variant was identified. In the local cases designated as NF2, 2% (0.5%) had an LZTR1 mutation, 0% (0%) had SMARCB1, 52% (49.5%) had a full constitutional NF2 pathogenic variant and in 21% (35%) no pathogenic variant was identified.

In terms of clinical features NF2 had a significantly lower mean life expectancy of 66.2 years compared to 76.9 for schwannomatosis. Apart from the expected excess of VS in people with NF2, they also had significantly more non-vestibular cranial schwannomas than people with schwannomatosis. However, people with schwannomatosis had more peripheral and spinal nerve schwannomas and pain was a predominant presenting symptom.

*Comment* Generating confident epidemiological statistics such as these are difficult to accomplish in very low frequency disease. The size of the cohorts in this study provide useful clinical and genetic insights into both schwannomatosis and NF2 as well as an important caution about the clinical overlap between these conditions. However, analysis of both national and regional patient groups does provide some variation that at times is difficult to interpret and the data presented would benefit from confidence intervals. Lastly, the large gap in precise genetic diagnoses in these conditions is highlighted with no pathogenic mutation identified in a considerable number of people who were clinically labelled as having NF2 or schwannomatosis.

*J Neurol Neurosurg Psychiatry. 2018 Jun 16. [Epub ahead of print]*

## Activity of selumetinib in neurofibromatosis type 1-related plexiform neurofibromas

The majority of patients with NF1 develop central and peripheral nerve sheath tumours caused by a lack of functioning neurofibromin which leads to dysregulation of Ras proteins (a family of proteins controlling cell signalling pathways and tumourigenesis). The development of plexiform neurofibromas in 20–50% of people with NF1 is attributed to elevated Ras-MAPK signalling. These tumours can cause significant complications including physical deformities, functional decline, pain, malignant transformation as well as cognitive and behavioural changes. At present, no effective medical treatment is available for plexiform neuromas and clinical management is largely centred around surveillance and symptom-guided surgical intervention, with complete resection frequently unfeasible. The advent of agents that target Ras signalling has opened up a potential therapeutic opportunity for NF1.

This study was a phase 1 trial of selumetinib, an oral selective inhibitor of MAPK kinase (MAP2K or MEK) 1 and 2. Selumetinib mitigates the downstream effects of up-regulation of Ras activity due to *NF1* mutations, and has already shown activity against advanced thyroid cancer, NSCLC and melanomas in adults in separate trials.

Twenty-four children aged 3–18 years (median 10.9) with a clinical diagnosis of NF1 and who had inoperable, measurable, plexiform neurofibromas that had potential to cause significant complications were included in the trial. Selumetinib (10 mg and 25 mg capsules) was administered approximately every 12 h on a continuous dosing schedule in 28-day cycles. Dose was calculated using body-surface area, starting with 20 mg per square metre and up to 50 mg per square metre. Patient assessments included a range of clinical examinations, laboratory evaluations, ophthalmologic examinations, echocardiography, and electrocardiography at baseline and at regular intervals. MRI images of tumours were obtained with volumetric analysis at baseline, after cycles 5 and 10, and then after every 6 subsequent cycles. The number of cycles of selumetinib administered ranged from 6 to 56 (median 30);19 patients received more than 30 cycles.

Selumetinib was well tolerated up to 25 mg per square metre every 12 h when taken over the long-term (median 120 weeks). Common side-effects included acneiform rash, gastro-intestinal upset and asymptomatic creatine kinase elevation. The most common dose-limiting toxic effects were mucositis, aminotransferase elevation, decreased neutrophil count, and paronychia. A reversible dose-dependent decrease in left ventricular function was seen in one patient. Five patients withdrew from treatment, 1 as a result of dose-limiting toxic effects.

Partial response, defined by a tumour volume decrease of ≥ 20% from baseline, was observed in 17 of the 24 children (71%). Disease progression defined as a ≥ 20% volume increase from baseline was not observed. Data on additional clinical benefits were not routinely collected, however, anecdotal evidence suggested that individual patients benefited from reductions in pain, degree of disfigurement and motor function. A parallel pharmacokinetic and pre-clinical study undertaken in a murine model of NF1 demonstrated a decrease in neurofibroma volume in 12 of 18 NF1 mice (67%) versus an increase of neurofibroma volume in 14 of the 15 control animals.

*Comment* These are preliminary results and require validation in larger studies, which should include qualitative analysis of any clinical improvements resulting from volumetric reduction of neurofibromas. However, these data indicate the potential for effective short- to medium-term tumour control in a disease which has hitherto been extremely difficult to manage and highly distressing for patients.

*N Engl J Med. 2016 Dec 29;375(26):2550–2560*

